# Swim‐up method is superior to density gradient centrifugation for preserving sperm DNA integrity during sperm processing

**DOI:** 10.1002/rmb2.12562

**Published:** 2024-01-29

**Authors:** Kenji Amano, Satoko Oigawa, Koichiro Ichizawa, Yukiko Tokuda, Mami Unagami, Mami Sekiguchi, Mayuko Furui, Kentaro Nakaoka, Ayumu Ito, Rika Hayashi, Yuko Tamaki, Yuko Hayashi, Yusuke Fukuda, Yukiko Katagiri, Masahiko Nakata, Koichi Nagao

**Affiliations:** ^1^ Reproduction Center, Toho University Omori Medical Center Tokyo Japan; ^2^ Department of Obstetrics and Gynecology, Faculty of Medicine Toho University Tokyo Japan; ^3^ Department of Urology, Faculty of Medicine Toho University Tokyo Japan

**Keywords:** density gradient centrifugation, in situ nick‐end labeling, sperm DNA fragmentation, sperm progressive motility, swim‐up

## Abstract

**Purpose:**

This study aimed to evaluate the effects of swim‐up and density gradient centrifugation methods on sperm DNA fragmentation.

**Methods:**

Nineteen normozoospermic patient samples with ≥100 × 10^6^ motile sperms were included in this study. Sperm DNA fragmentation, progressive motility, and progressive motile sperm number were measured before and after the swim‐up method or density gradient centrifugation.

**Results:**

Sperm DNA fragmentation was not statistically different between swim‐up—(14.4 ± 2.1%, *p* = 0.32) and density gradient centrifugation‐processed (25.0 ± 3.0%, *p* = 0.20) and unprocessed semen samples (19.2 ± 1.9%). Sperm DNA fragmentation was significantly lower in swim‐up—than in density gradient centrifugation‐processed samples (*p* < 0.05). Sperm progressive motility was significantly higher (*p* < 0.05) in swim‐up—(92.9 ± 1.0%) and density gradient centrifugation‐processed (81.3 ± 2.0%) samples, with the former being higher, than in unprocessed semen samples (53.1 ± 3.7%). The recovery rate of progressive motile sperms was significantly lower in swim‐up—(9.7 ± 1.4%) than in density gradient centrifugation‐processed samples (17.2 ± 1.8%, *p* < 0.05).

**Conclusions:**

The swim‐up method is superior to density gradient centrifugation, evidenced by less sperm DNA fragmentation and higher sperm progressive motility. The recovery rate of progressive motile sperms was better after density gradient centrifugation than after swim‐up.

## 1 INTRODUCTION

Infertility is defined as failure to achieve pregnancy after 12 months of regular unprotected sexual intercourse.^1^ Approximately one in 5.5 couples in Japan currently experiences fertility problems, and approximately one in 14 fetuses is born using assisted reproductive technology (ART). Approximately 50% of infertility cases are attributed to factors of male origin.^2^ Most of these are spermatogenic dysfunctions, including decreased sperm number and motility. These dysfunctions can be relatively easily evaluated; however, recently, abnormalities at the molecular level, such as sperm DNA fragmentation, are being considered important for male infertility. Oleszczuk et al.^3^ reported a negative association between increased sperm DNA fragmentation (more than 20%) and good‐quality embryos and live birth rates in in vitro fertilization (IVF). Wdowiak et al.^4^ found that a low sperm DNA fragmentation index (the percentage of spermatozoa with DNA lesions) corresponds to an increased rate of blastocyst development and positive pregnancy outcomes following intracytoplasmic sperm injection (ICSI). Sperm DNA fragmentation can be caused by several factors, including oxidative stress^5^, ^6^ and centrifugation, the latter being used to purify and wash sperms for IVF or artificial insemination.^7^ Therefore, assessing the effect of different sperm preparation methods on sperm DNA fragmentation to optimize sperm preparation for IVF is important.

Sperm DNA fragmentation can be measured using four main procedures: sperm chromatin structure assay (SCSA),^8^ terminal deoxynucleotidyl transferase‐mediated digoxigenin‐UTP nick‐end labeling (TUNEL),^9^ sperm chromatin dispersion (SCD),^10^ and comet assay. TUNEL and comet assays directly assess the presence of single‐ or double‐stranded breaks in DNA. In contrast, SCSA and SCD assays detect the susceptibility of chromatin to acid treatment and indirectly assess DNA fragmentation. Furthermore, the TUNEL assay coupled with flow cytometry provides reliable data.^11^

Swim‐up and density gradient centrifugation are the most commonly used methods for semen preparation. Although several reports have assessed sperm DNA fragmentation after swim‐up or density gradient centrifugation procedures, the results have been inconsistent.^12^, ^13^, ^14^, ^15^, ^16^, ^17^, ^18^ This study aimed to compare sperm DNA fragmentation between swim‐up and density gradient centrifugation methods using the TUNEL coupled with flow cytometry procedure.

## 2 MATERIALS AND METHODS

### 2.1 Sample collection

Human sperm samples were obtained from 19 normozoospermic patients who sought medical consultation for semen analysis between November 2021 and September 2022. The median age of the patients was 36 years (range, 28–53 years). Samples with a total of ≥100 × 10^6^ motile sperms were included in the study because sperm DNA fragmentation analysis using the TUNEL method with flow cytometry requires 1–2 × 10^6^ sperms after the swim‐up or density gradient centrifugation procedure as per the kit manufacturer's instructions. This study was approved by the Review Board of Toho University Omori Medical Center (approval no. M22015 20339 20048). Written informed consent was obtained from all patients. Semen samples were collected via masturbation. Sperm motility and concentration were measured using a sperm motility analysis system (DITECT Co., Ltd., Tokyo, Japan). Progressive motility and the total number of sperms with progressive motility were measured. The recovery rate of progressive motile sperm was calculated as follows: recovery rate = total number of sperms with progressive motility after swim‐up or density gradient centrifugation/half of the total number of sperms with progressive motility of raw semen × 100. Semen volume was measured using a 5‐mL plastic syringe with an 18‐gauge flat needle.

### 2.2 Swim‐up method

Half the volume of the semen samples was washed with 4 mL of 4‐(2‐hydroxyethyl)‐1‐piperazineethanesulfonic acid (HEPES) medium (pH 7.3) (Nakamedical Inc., Tokyo, Japan). The samples were then centrifuged at 400 × *g* for 5 min. The upper part of the supernatant was removed, and the sperm pellet was resuspended in the remaining 0.5 mL of HEPES medium. The resuspended pellet was submerged in 1 mL of HEPES medium and incubated for 1 h at 37°C at an angle of 45 degrees. Next, actively motile sperm with 0.6–0.9 mL of supernatant was removed and placed in a new tube.

### 2.3 Density gradient centrifugation

The other half volume of the semen sample was layered on top of the density gradient (80% SepaSperm® Solution, Kitazato Corp., Tokyo, Japan) and centrifuged at 400 × *g* for 20 min at 20–25°C. The sperm pellet was washed with 4 mL of HEPES medium and centrifuged at 400 × *g* for 5 min. The supernatant was removed, and the remaining 0.5 mL of HEPES medium and the sperm pellet were resuspended.

### 2.4 Measurement of sperm DNA fragmentation

Sperm DNA fragmentation was evaluated by the TUNEL assay using an Apo‐Direct™ Apoptosis Detection Kit (Thermo Fisher Scientific Inc., Waltham, CA, USA) and a flow cytometer (BD FACS Canto‐II; BD Biosciences, San Jose, CA, USA).

Briefly, raw semen samples after swim‐up and after density gradient centrifugation procedures, containing 1–2 × 10^6^ spermatozoa, which were measured using a sperm motility analysis system, were fixed in 1% w/v paraformaldehyde in phosphate‐buffered saline (PBS, pH 7.4) for 45 min on ice. The spermatozoa were centrifuged at 1000 × *g* for 5 min, and the sperm pellets were washed with 1 mL of PBS. The washed sperm pellets were resuspended in 1 mL of PBS and stored in a refrigerator until sperm DNA fragmentation.

The stored sperm samples were left at 20–25°C for approximately 5 min and washed twice with 1 mL of wash buffer. Next, 50 μL of the DNA labeling solution containing FITC‐dUTP and terminal deoxynucleotidyl transferase (TdT) enzyme was added, and the mixture was incubated for 1 h at 37°C. The samples were then washed twice with 1 mL of rinse buffer. The samples were resuspended in 0.5 mL of propidium iodide/RNase A solution and incubated in the dark for 30 min at 20–25°C. Sperm DNA fragmentation was analyzed using a BD FACS Canto‐II flow cytometer with a 488‐nm argon laser (BD Biosciences). In total, 10 000 events were recorded for each sample. A positive control was prepared by a prior 0.02% hydrogen peroxide treatment for 50 min at 20–25°C to fragment sperm DNA before fixation with 1% w/v paraformaldehyde in PBS. A negative control was prepared as a DNA labeling solution without the TdT enzyme.

### 2.5 Statistical analyses

All statistical analyses were performed using EZR (Saitama Medical Center, Jichi Medical University, Saitama, Japan), a graphical user interface for R (The R Foundation for Statistical Computing, Vienna, Austria, version 4.2.2).^19^ This is a modified version of the R commander designed to add statistical functions frequently used in biostatistics. Continuous data were checked for normality using the Shapiro–Wilk test. Normally distributed continuous data were compared using analysis of variance (ANOVA), and non‐normally distributed continuous data were compared using the Wilcoxon signed‐rank test. Subsequently, a multi‐comparison Tukey's range test was used if *p* < 0.05. A *p*‐value < 0.05 indicated significant differences (*p < 0.05). Data are presented as the mean ± standard error of the mean (SEM). Linear regression analysis was used to investigate any association between sperm DNA fragmentation and progressive motility in swim‐up and density gradient centrifugation samples.

## 3 RESULTS

### 3.1 Basic semen characteristics of 19 normozoospermic patients

The characteristics of raw semen samples obtained from 19 normozoospermic patients were as follows (median [range]): semen volume, 2.6 mL (1.2–7.0 mL); sperm count, 82.0 million/mL (27.4–230.9 million/mL); total motility, 70.8% (34.5%–93.8%); total motile sperm count, 126.8 million (100.4–495.7 million); and percentage of normal form, 7.0% (4.0%–13.0%).

### 3.2 Evaluation of sperm DNA fragmentation

**FIGURE 1:**
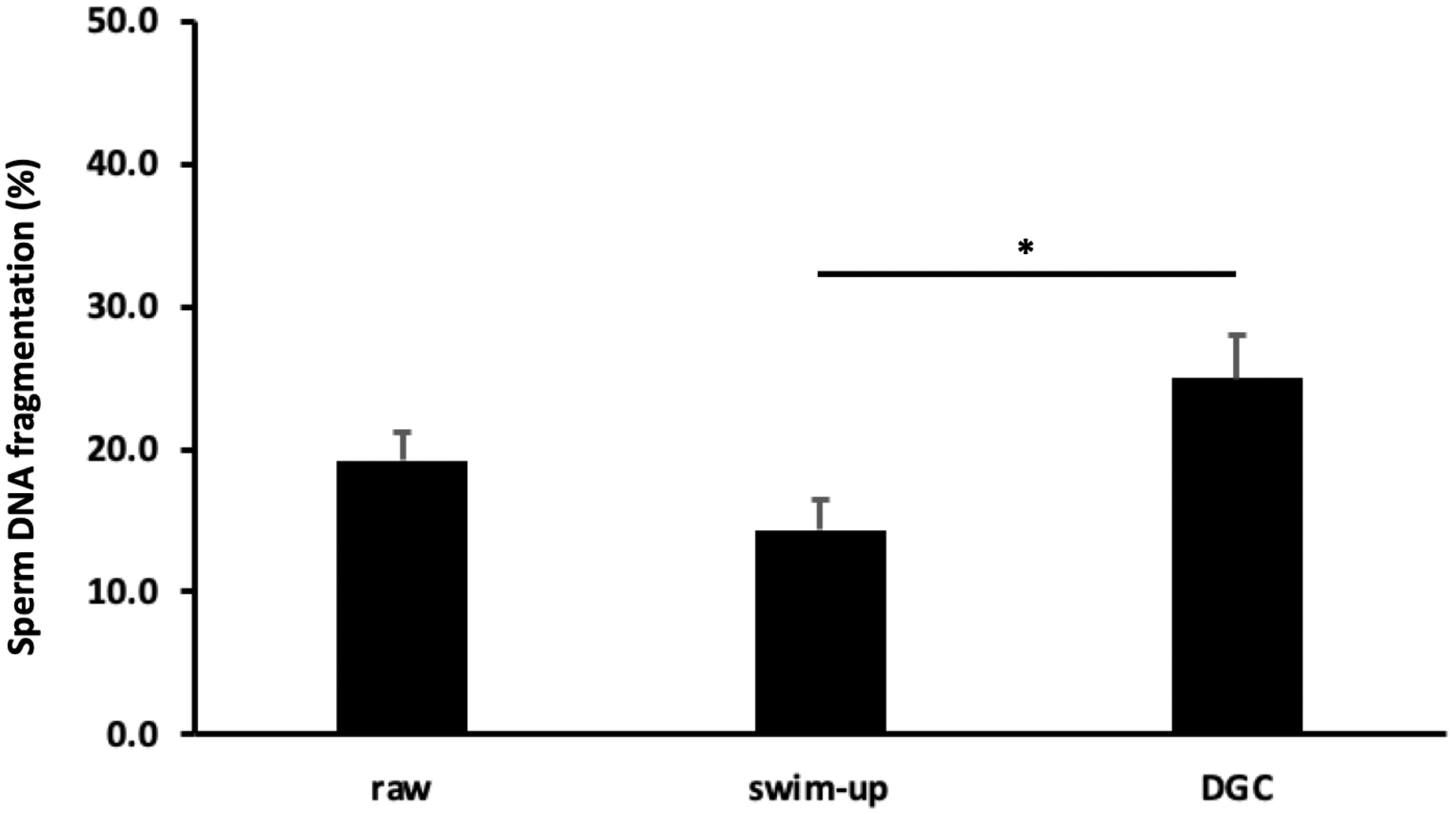
Sperm DNA fragmentation among raw semen, swim‐up, and density gradient centrifugation samples. *N* = 19 for each sample. ANOVA and Tukey's range test were used for statistical analyses. *p < 0.05 indicates statistical significance. Data are presented as the mean ± SEM. DGC, density gradient centrifugation.

The DNA fragmentation percentage was 19.2 ± 1.9% (5.3%–37.9%) in raw semen samples (Figure 1), which did not significantly differ from that in swim‐up samples [14.4 ± 2.1% (3.5%–37.1%), *p* = 0.32] and density gradient centrifugation samples [25.0 ± 3.0% (3.1%–51.1%), *p* = 0.20]. Notably, the DNA fragmentation percentage was significantly lower in swim‐up samples than in density gradient centrifugation samples (*p* < 0.05, Tukey's range test).

### 3.3 Evaluation of sperm progressive motility

**FIGURE 2:**
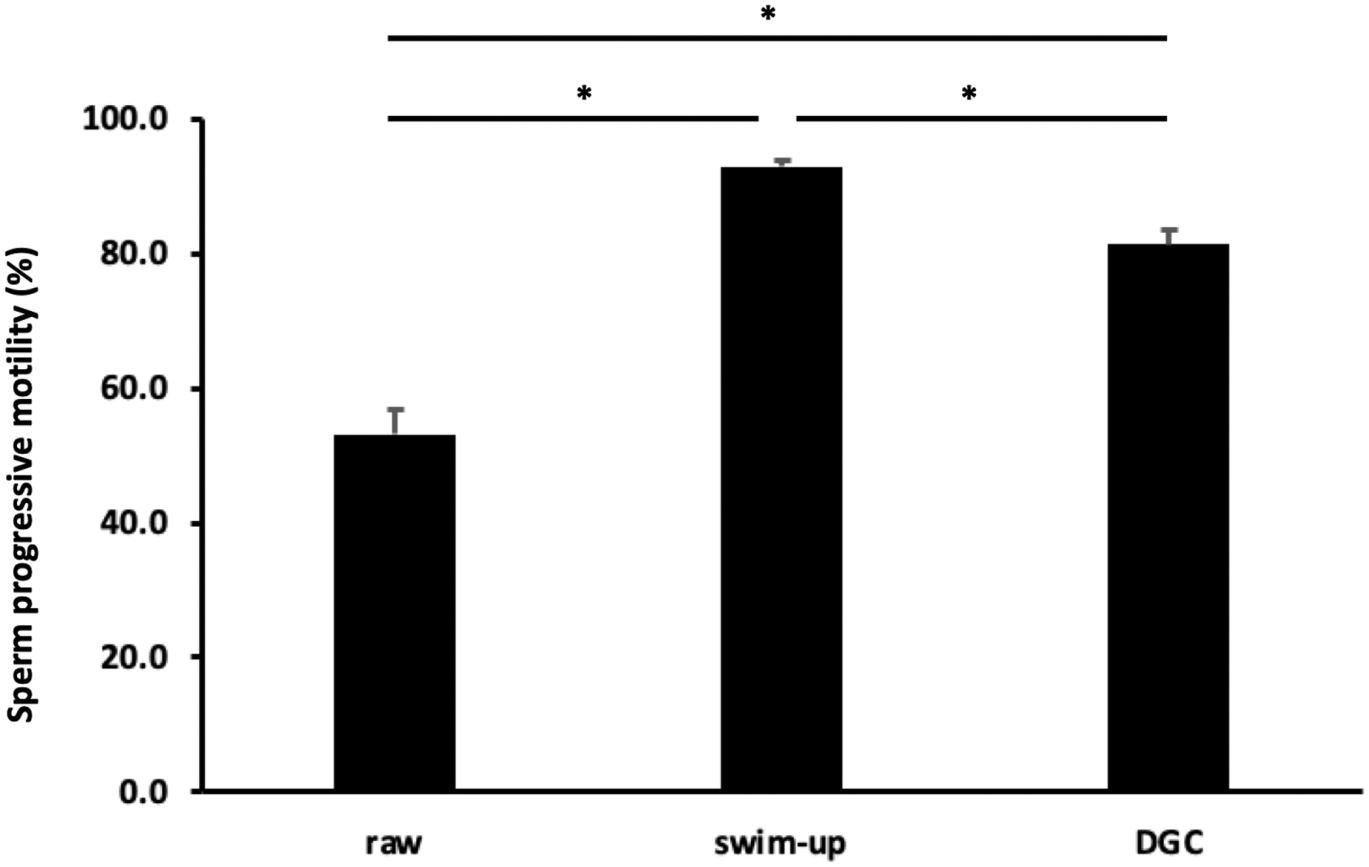
Sperm progressive motility among raw semen, swim‐up, and density gradient centrifugation samples. *N* = 19 for each sample. ANOVA and Tukey's range test were used for statistical analyses. *p < 0.05 indicates statistical significance. Data are presented as the mean ± SEM. DGC, density gradient centrifugation.

Sperm progressive motility was 53.1 ± 3.7% (26.1%–80.7%) in raw semen samples (Figure 2). A significantly higher sperm progressive motility was found in swim‐up—processed [92.9 ± 1.0% (84.7%–100.0%), *p* < 0.05; Tukey's range test] and density gradient centrifugation‐processed [81.3 ± 2.0% (58.8%–94.3%), *p* < 0.05; Tukey's range test] samples than in the raw semen samples. Furthermore, sperm progressive motility was significantly higher in the swim‐up sample than in the density gradient centrifugation sample (*p* < 0.05; Tukey's range test).

### 3.4 Evaluation of the recovery rate of progressive motile sperms

**FIGURE 3:**
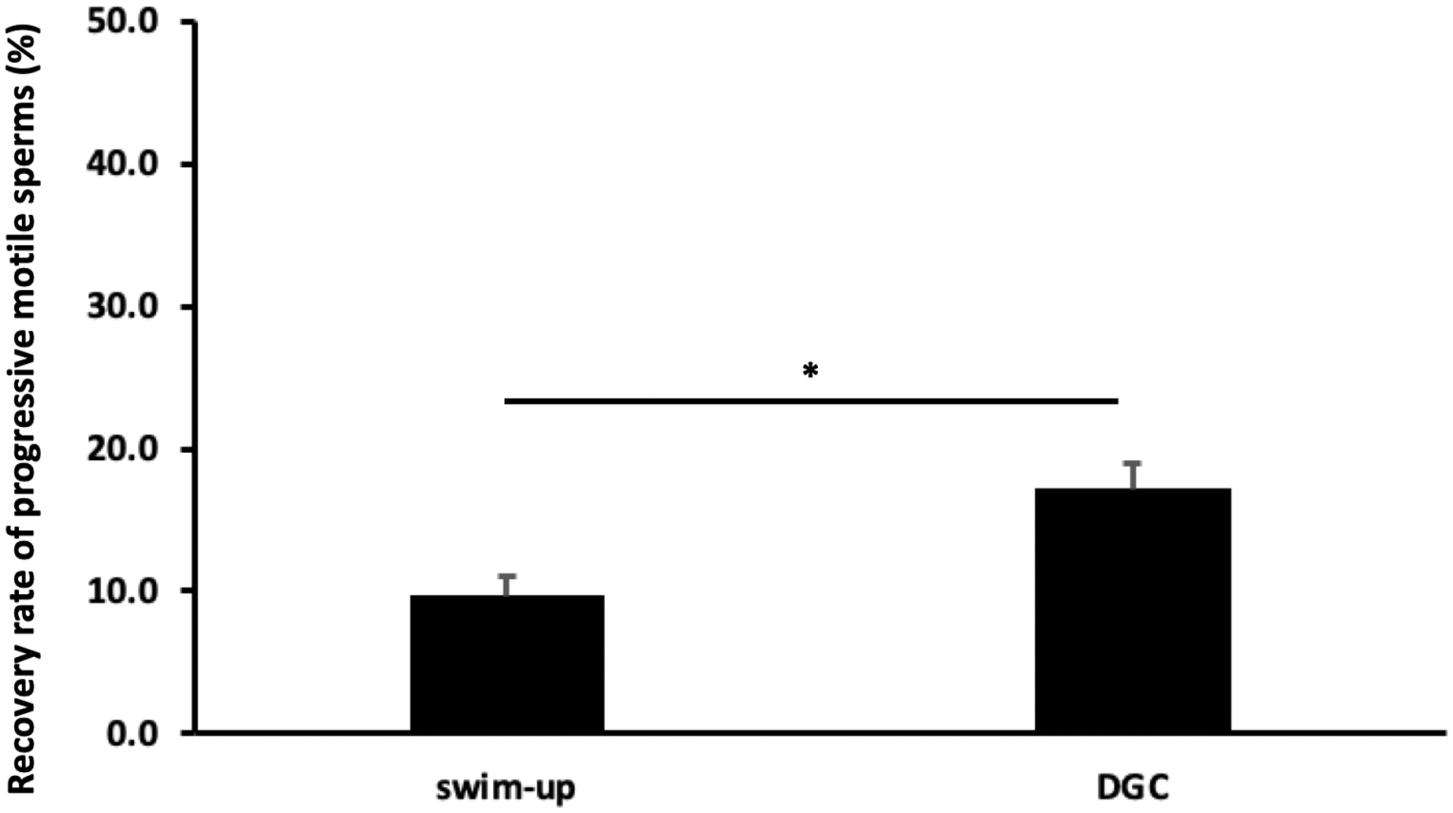
Recovery rate of progressive motile sperms after swim‐up or density gradient centrifugation. *N* = 19 for each sample. Wilcoxon signed‐rank test was used for statistical analysis. *p < 0.05 indicates statistical significance. Data are presented as the mean ± SEM. DGC, density gradient centrifugation.

The recovery rate of progressive motile sperms in the swim‐up and density gradient centrifugation samples was 9.7 ± 1.4% (2.89%–23.37%) and 17.2 ± 1.8% (6.53%–34.02%, *p* < 0.05; Wilcoxon signed‐rank test), respectively (Figure 3).

### 3.5 Linear regression analysis of sperm DNA fragmentation and progressive motility

**FIGURE 4:**
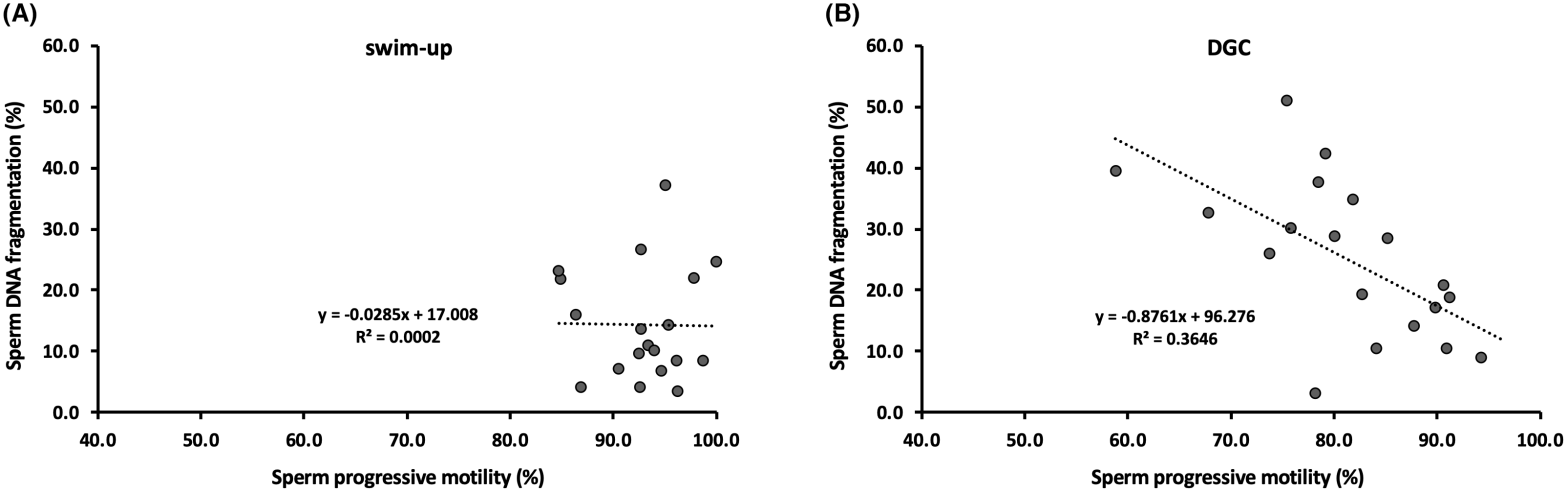
Scatterplots of sperm DNA fragmentation and progressive motility after swim‐up (A) or density gradient centrifugation (B). *N* = 19 for each sample. Linear regression analysis was used for statistical analysis. DGC, density gradient centrifugation.

We plotted the relationship between sperm DNA fragmentation and progressive motility in swim‐up‐processed and density gradient centrifugation‐processed samples (Figure 4). The swim‐up‐processed samples appeared to cluster toward high progressive motility and low sperm DNA fragmentation values, without significant correlation (coefficient − 0.06, *p* = 0.95; Figure 4A). In contrast, for the density gradient centrifugation‐processed samples, increased progressive motility was related to decreased sperm DNA fragmentation, while some samples also had relatively low progressive motility and high sperm DNA fragmentation (coefficient − 3.12, *p* < 0.05; Figure 4B).

## 4 DISCUSSION

In the present study, we compared sperm DNA fragmentation after processing using two different procedures, swim‐up and density gradient centrifugation, employing the TUNEL assay coupled with flow cytometry. Sperm DNA fragmentation was significantly lower after the swim‐up method than after density gradient centrifugation.

There are several reports on DNA fragmentation of sperm using different sperm preparation methods; however, the results are inconsistent.^12^, ^13^, ^14^, ^15^, ^16^, ^17^, ^18^ Jayaraman et al. performed a TUNEL assay in sperm from normozoospermia, oligozoospermia, and teratozoospermia patients after sperm processing (density gradient centrifugation, swim‐up, or density gradient centrifugation followed by swim‐up) and found that the percentage of TUNEL‐positive sperms was significantly lower in each sample after sperm processing.^12^ Moreover, no differences were observed in the incidence of TUNEL‐positive sperms among the various techniques.^12^ Amiri et al. analyzed sperm DNA fragmentation using the comet assay and found that density gradient centrifugation was superior to the swim‐up method.^13^ Xue et al.^14^ used the SCD method to measure sperm DNA fragmentation in teratozoospermia and found that both swim‐up‐ and density gradient centrifugation‐processed samples had significantly lower sperm DNA fragmentation than unprocessed sperm, where density gradient centrifugation was found to be better than swim‐up. In contrast, Volpes et al.^15^ measured sperm DNA fragmentation using the SCD method after using four preparation techniques (direct swim‐up, pellet swim‐up, density gradient centrifugation, and density gradient centrifugation followed by swim‐up). Sperm DNA fragmentation in all processed samples was found to be significantly lower than that in raw semen, and the value in the swim‐up method was superior to that in density gradient centrifugation.^15^ Raad et al.^16^ found similar results; sperm DNA fragmentation was significantly lower in semen swim‐up and pellet swim‐up samples than in density gradient centrifugation or density gradient centrifugation followed by swim‐up samples. Two other studies reported that sperm DNA fragmentation in swim‐up was significantly lower than that in density gradient centrifugation.^17^, ^18^ In these reports, sperm DNA fragmentation in density gradient centrifugation was almost similar to that in raw semen.^17^, ^18^ These discrepancies might be attributed to the different samples analyzed, methods used, or institutions. However, most of the recently published studies showed superiority of the swim‐up method over density gradient centrifugation. Our results also indicated significantly less sperm DNA fragmentation in the swim‐up method than in density gradient centrifugation, and the value of density gradient centrifugation was almost the same as that of raw semen. In addition, regression of sperm DNA fragmentation against progressive motility suggested that more sperm with low DNA fragmentation was collected in the swim‐up‐processed samples than in the density gradient centrifugation‐processed samples. In our study, centrifugation was performed twice (400 × *g*, 20 min and 400 × *g*, 5 min) during density gradient centrifugation and once (400 × *g*, 5 min) during swim‐up. Therefore, long‐term and repeated centrifugation procedures could negatively affect sperm DNA fragmentation. Moreover, the swim‐up method does not require any additional devices or media and reduces the cost as compared to density gradient centrifugation.

Concerning the methodology for sperm DNA fragmentation, we used the TUNEL assay coupled with flow cytometry, and the target sperms were approximately 2000 propidium iodide‐positive sperms. The TUNEL method directly assesses DNA fragmentation by labeling the 3′ hydroxyl break‐ends of ssDNA and dsDNA. The comet assay also directly assesses DNA fragmentation as having a comet‐like profile when DNA embedded in agarose gel is electrophoresed. In contrast, the SCD test indirectly assesses DNA fragmentation by analyzing the characteristic halo of dispersed DNA loops observed in sperm with non‐fragmented DNA following acid denaturation and removal of nuclear proteins.^10^ Jayaraman et al.^12^ performed TUNEL assays but assessed 500 sperms on slides. The comet assay and SCD test were also performed on slides, and the number of target sperms was approximately 500.^13^, ^14^, ^15^, ^16^, ^17^, ^18^ Therefore, the TUNEL method coupled with flow cytometry provides direct and massive data on sperm DNA fragmentation.

The value of progressive motile sperms was significantly higher in the swim‐up than in the density gradient centrifugation samples. The density gradient centrifugation method utilizes the difference in specific gravity between mature and immature sperms. Therefore, sperms collected by density gradient centrifugation are mature and motile. However, some immature or immotile sperms may be separated in this process as well, reducing the motility of the collected sperm.

The recovery rate of progressive motile sperm was significantly lower in the swim‐up than in the density gradient centrifugation samples. The swim‐up method recovers only motile sperm, whereas the density gradient centrifugation method may recover immature and immotile sperm because of the slight difference in the specific gravity between immature and mature sperm. However, the recovery rate of progressive motile sperms in the swim‐up sample was approximately 10% (10 × 10^6^ motile sperm) of that in the raw semen sample (100 × 10^6^ motile sperm), and the number of progressive motile sperms was sufficient to perform IVF (0.1 × 10^6^ motile sperm) or ICSI.

In this study, we used normozoospermic patients with a total number of motile sperms ≥100 × 10^6^ because TUNEL coupled with flow cytometry requires 1–2 × 10^6^ sperms after the swim‐up or density gradient centrifugation procedure. It was somehow difficult to collect samples because some patients who went to an infertility clinic had some abnormalities in semen parameters.

In conclusion, our results suggest the superiority of the swim‐up method over the density gradient centrifugation method in term of sperm DNA fragmentation and sperm progressive motility. To the best of our knowledge, this is the first study to compare sperm DNA fragmentation using a TUNEL assay coupled with flow cytometry. Our data provide useful insights into sperm preparation methods for ART.

## CONFLICT OF INTEREST STATEMENT

The authors declare that there are no conflict of interest related to this article.

## ETHICS STATEMENT

This study was approved by the Review Board of Toho University Omori Medical Center (protocol number: M22015 20339 20048).

## HUMAN RIGHTS STATEMENTS AND INFORMED CONSENT

All procedures were performed following the ethical standards of the relevant committees on human experimentation (institutional and national) and the Helsinki Declaration of 1964 and its later amendments. Informed consent was obtained from all patients included in the study.
